# A Global Perspective on H9N2 Avian Influenza Virus

**DOI:** 10.3390/v11070620

**Published:** 2019-07-05

**Authors:** T(homas). P. Peacock, Joe James, Joshua E. Sealy, Munir Iqbal

**Affiliations:** 1 Avian Influenza Group, The Pirbright Institute, Woking GU24 0NF, UK; 2 Section of Virology, Faculty of Medicine, Imperial College London, London W2 1PG, UK; 3 Royal Veterinary College, London NW1 0TU, UK

**Keywords:** H9N2, avian influenza viruses, zoonotic, pandemic potential, poultry

## Abstract

H9N2 avian influenza viruses have become globally widespread in poultry over the last two decades and represent a genuine threat both to the global poultry industry but also humans through their high rates of zoonotic infection and pandemic potential. H9N2 viruses are generally hyperendemic in affected countries and have been found in poultry in many new regions in recent years. In this review, we examine the current global spread of H9N2 avian influenza viruses as well as their host range, tropism, transmission routes and the risk posed by these viruses to human health.

## 1. Introduction

Influenza A viruses are members of the *Orthomyxoviridae* family and contain a segmented, negative-sense RNA genome encoding 10 core proteins and a variable number of accessory proteins. Influenza A viruses are commonly characterised by their combinations of surface proteins, haemagglutinin (HA) and neuraminidase (NA), giving rise to a multitude of different subtypes designated, for example, as H1N1, H5N6, or H9N2.

The natural host of influenza viruses are wild waterfowl and sea birds which contain almost every known subtype of influenza (with the exceptions of H17N10 and H18N11 which have only been found in bats) [1]. Viruses sporadically and periodically spill over from wild bird hosts to infect domestic poultry. Generally, these viruses circulate briefly before dying out (either naturally or by human interventions such as biosecurity and vaccination), for example the repeated incursions of H7Nx viruses into Europe and North America during the 1990s and 2000s [2,3,4]. Occasionally, however, a lineage of avian influenza will become well-adapted to poultry and continue circulating endemically, for example, the panzootic goose/Guangdong lineage H5Nx viruses, the recent Chinese H7N9 viruses, and multiple Eurasian H9N2 lineages [5,6,7].

Avian influenza viruses (AIVs) can be broadly categorised into two groups based on a combination of their pathogenicity in chickens and molecular markers in their HA protein. Highly pathogenic avian influenza viruses (HPAIV) display high pathogenicity in chickens (when tested using an intra-venous pathogenicity index; IVPI) and contain polybasic cleavage sites in HA, resulting in the protein being cleaved by endogenous cellular furin-like proteases, allowing the virus to replicate systemically in birds. Only the H5 and H7 subtypes have ever shown this phenotype in the field with examples of HPAIV including goose/Guangdong-lineage H5Nx viruses, sporadic H7Nx outbreaks, and recent H7N9 viruses. Low pathogenicity avian influenza viruses (LPAIVs) are characterised by low pathogenicity in chickens (as measured by IVPI) and mono- di- or occasionally tri-basic cleavage sites in haemagglutinin, these only allow cleavage of HA by extracellular trypsin-like proteases restricting the virus largely to the respiratory and gastrointestinal tracts, where such proteases are abundantly expressed.

H9N2 viruses, the topic of this review, are an LPAIV subtype found worldwide in wild birds and are endemic in poultry in many areas of Eurasia and Africa. Compared to H5 and H7 viruses they are somewhat neglected, however, recent evidence, summarised in this review, suggests they could potentially have a major role in the emergence of the next influenza pandemic, either directly as an H9N2 subtype virus, or through the donation of internal genes to a pandemic virus.

## 2. History and Phylogeography of H9N2 Virus in Poultry

**Figure 1:**
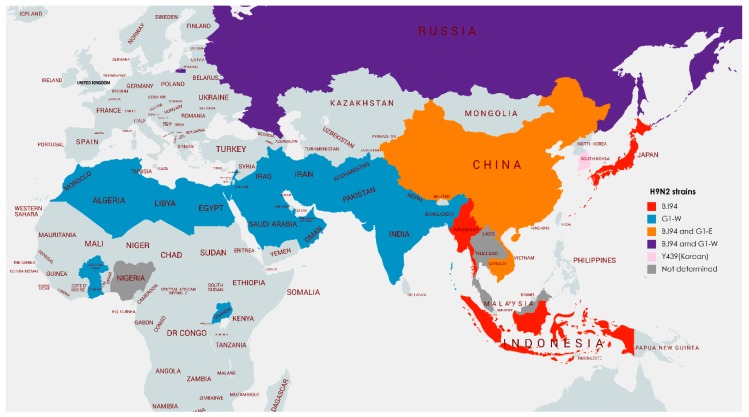
Phylogeographic range of poultry-adapted H9N2 lineages. Countries where only BJ94 lineage viruses are found shown in red, where only G1-W viruses found shown in blue, where mixtures of BJ94 and G1-E sub-lineage viruses are found shown in orange, where mixtures of BJ94 and G1-W sub-lineage viruses are found shown in purple, where only poultry-adapted Y439-lineage viruses are found shown in light pink. H9N2-positive countries where H9N2 lineage hasn’t been determined shown in grey. Figure made using mapchart.net.

H9N2 viruses were first isolated from turkeys in the US state of Wisconsin in 1966 [8]. In the following decades, the virus was occasionally isolated during sporadic outbreaks in poultry in the Northern USA, and from wild birds and domestic ducks throughout Eurasia [9]. In the early 1990s, the virus was first isolated from chickens in China and in the following decades viruses related to this Chinese progenitor have become endemic in farmed poultry across much of Asia, the Middle East and North and West Africa [10] (see Figure 1).

H9N2 viruses are often found co-circulating in poultry with other AIV subtypes, such as H5 and H7 HPAIVs. There is good evidence to suggest that prior or concurrent H9N2 infection can mask the high mortality rate due to these viruses allowing ‘silent’ spread of HPAIVs, thwarting surveillance and subsequent intervention efforts [11,12].

### 2.1. Phylogeography of H9N2 Viruses

Phylogenetically, the HA gene of H9N2 viruses can be broadly split into two major branches, a Eurasian branch and an American branch. American H9N2 viruses are mostly found in wild birds but have been described to infect farmed turkeys without stably circulating in poultry. Eurasian H9N2 viruses, conversely, have established at least three stable poultry lineages, named after their prototypic viruses, A/quail/Hong Kong/G1/1997, A/chicken/Beijing/1/94 and A/chicken/Hong Kong/Y439/1997, known consequently as the G1, BJ94 (also known variously as the Y280 or G9 lineage) and Y439 (also sometimes known as the Korean lineage) lineages [5]. The G1 lineage can further be split into two phylogenetic and geographical sub-lineages referred to as the ‘Western’ and ‘Eastern’ sub-lineages. H9N2 lineage and sub-lineages can be, and routinely are, further subdivided based on relatedness and geographical distribution [13,14,15,16], however for this review the previously described lineages/sub-lineages will be used throughout. 

Global surveillance of LPAIV, such as H9N2, has a problem when compared to HPAIV viruses in that LPAIV H9N2 is not a notifiable pathogen and causes relatively few overt human infections. In many resource-limited regions surveillance is performed sporadically, or not at all. It is likely that H9N2 viruses are present or even endemic in more countries, particularly in low- and middle-income countries in Africa and Asia, than is outlined below. For example, poultry adapted strains of the virus usually spread short distances (rather than by long distance flyways), therefore the isolation of the virus in Uganda in West Africa, most related (though not very closely related) to viruses from the Arabian Peninsula, ~2000km away, suggests that it is likely countries in-between also contain intermediately related H9N2 viruses which are yet to be isolated [17].

#### 2.1.1. East and Southeast Asia

H9N2 viruses are considered endemic in China, Vietnam and South Korea (see Table 1, Figure 1) [5,18,19,20]. In recent years, the virus has been isolated for the first time in Cambodia, Myanmar, Indonesia, Malaysia and the Russian Far East and serological evidence suggests the virus may also be present in poultry in Laos and Thailand [21,22,23,24,25,26,27,28]. BJ94 lineage viruses are found throughout China, Vietnam, Cambodia, Myanmar and Indonesia. G1 ‘Eastern’ viruses are also found in South China, Vietnam and Cambodia, mostly infecting minor poultry species such as quail. Y439 lineage viruses have been found in wild birds (and sporadically in poultry) throughout Eurasia but a distinct poultry-adapted subset circulates endemically in poultry in South Korea. Vaccination of poultry has been used in recent years to try to control endemic diseases in large areas of China and South Korea [29,30].

#### 2.1.2. South Asia

H9N2 viruses are considered endemic in Bangladesh and Pakistan and are likely endemic in regions of India, Afghanistan and Nepal [20,34,35,36,37,38,39]. G1 ‘Western’ viruses constitute the majority of viruses found in poultry in South Asia, with a few Y439 viruses occasionally spilling over into poultry from wild birds (but apparently not maintaining sustained transmission). The predominant G1 ‘Western’ sub-lineage of viruses in this region (as well as in Iran) appears to have arisen from a reassortment event between co-circulating HPAIV H7N3 and LPAIV H9N2 viruses, which replaced other local clades [40,41].

#### 2.1.3. The Middle East

H9N2 is frequently isolated from, and therefore probably endemic in poultry in many Middle Eastern countries including Egypt, Iran, Israel, Saudi Arabia and the United Arab Emirates [14,20,34,38,42,43,44]. The virus has also been isolated regularly in Iraq, Jordan, Kuwait, Lebanon and Oman [39,45,46,47,48]. In Israel, mass vaccination of poultry, which began in 2003, has had some success in limiting the endemicity of the virus. This vaccine regime has necessitated an update of the vaccine seed strain at least once due to antigenic drift [49]. Extensive surveillance in Israel, between 2006 and 2012, has indicated that rather than there being a single locally evolving strain, viruses appear to be periodically eradicated, then reintroduced into the country. 

As with South Asia, the majority of H9N2 viruses found in the Middle East are of the G1 ‘Western’ sub-lineage, with occasional isolation of Y439 lineage viruses, likely originating from direct spillover events from wild birds.

#### 2.1.4. Africa

H9N2 viruses have been isolated from several African countries, the virus appears endemic in poultry in Egypt and has been repeatedly isolated from chickens in Libya and Tunisia [38,42,50,51]. Additionally, since 2016 the virus has been isolated for the first time in countries across North and West Africa including Morocco, Burkina Faso, Ghana and Algeria as well as in East Africa in Uganda [17,52,53,54,55]. Morocco has subsequently undertaken an apparently successful mass poultry vaccination programme [52]. All viruses isolated from poultry in Africa have been of the G1 ‘Western’ sub-lineage, related to those circulating in the Middle East in Israel, Jordan, Lebanon, Saudi Arabia and the United Arab Emirates.

H9N2 viruses have been isolated from farmed ostriches in South Africa on several occasions, however, due to their homology to wild bird virus isolates (of the Y439 lineage), and subsequent sampling that found no further evidence of circulation of the viruses, it appears these viruses most likely represent dead-end spillover events from wild migratory birds [56].

Finally, there are a pair of studies showing high seropositivity against H9N2 in Nigerian poultry and agricultural workers, however, no virus has been isolated from this country [57,58]. Although surveillance for HPAIVs is ongoing in Nigeria, it is unclear whether protocols are used that would pick up the presence (or absence) of H9N2 viruses, therefore it remains unclear whether the virus is/was present in this region.

As discussed previously, the presence of H9N2 virus in poultry across non-contiguous regions of Africa suggests that additional countries may harbour infection. However, there is no confirmation due to the virus not being actively surveyed for, or if found, not being reported due to LPAIs such as H9N2 infections not being diseases that are notifiable to the World Organisation for Animal Health (OIE).

#### 2.1.5. Europe

There is currently little evidence of endemic H9N2 in poultry in Europe, despite rigorous sampling (especially within the European Union). There is, however, good evidence for the virus in wild birds in Europe, mostly of the Y439 lineage, which occasionally spills over into farmed poultry (generally turkeys), for example in the UK, the Netherlands, Poland, Hungary, Italy and Ireland [20,38,59,60,61]. Germany appears to suffer from recurrent introduction of H9N2 viruses into its poultry from wild birds and there is even a single report of a G1 lineage H9N2 virus. Due to this continuous spillover autologous vaccines have been deployed in some regions [20,38,62].

Finally, there is a single study showing sero-prevalence of H9N2 antibodies in Romanian agriculture workers [63], similarly to the study from Nigeria, H9N2 virus has not been isolated from poultry in this country, therefore it remains to be seen if the virus is truly present here.

#### 2.1.6. The Americas

H9N2 viruses have been isolated from poultry in the USA periodically throughout the second half of the twentieth Century, in fact the prototypic H9N2 isolate (A/turkey/Wisconsin/1/1966) was isolated in this period. All isolated viruses have been of the American lineage and appear to be spillover events from wild birds, possibly sea birds which carry genetically closely related viruses in this region. Since 2001, there has been no evidence of the virus in poultry in North America, despite routine surveillance and extensive evidence of other non-H9N2 viruses in poultry [64,65,66,67,68]. 

In South America, there is serological evidence from 2005 of H9N2 infections in Colombia, however, no virus was isolated and no further evidence has been reporter since [66].

### 2.2. Hyper-Prevalence of H9N2 Viruses in Poultry

Whenever H9N2 virus prevalence has been investigated in lower- and middle-income countries, either by poultry sero-surveys or by passive sampling (i.e., random sampling of apparently healthy birds), the virus has been found to be present at extremely high rates, particularly in live bird markets (LBMs). LBMs act as hubs for poultry traders and their birds and are a major component of the disease transmission pathway, shown to maintain AIV dissemination among poultry as well as facilitate zoonotic infection [69,70]. In recent surveys in Vietnam, prevalence of the virus exceeded 3.5% in chickens in LBMs [71,72] and in various Chinese provinces, prevalence was found to be upwards of 10% [73,74,75,76]. Several separate studies have shown that the prevalence in Bangladesh and Pakistan of H9 viruses in chickens at LBMs and farms was almost 10% [35,77,78,79]. Another recent study has shown prevalence of upwards of 10% at LBMs in Egypt. Overall, these studies imply a degree of hyper-endemicity not seen for other influenza virus subtypes, potentially due to the LPAIV phenotype of the virus allowing repeated re-infections of the same birds (in the case of longer-lived layers and breeders) and silent spread between farms and smallholdings.

## 3. H9N2 Reassortment and Evolution

### 3.1. H9N2 Virus Pathogenesis

H9N2 viruses are nearly uniformly low pathogenicity in experimental settings when tested by IVPI [5,20,40], however, in the field they often exhibit moderate-to-high morbidity and mortality. For example, there are many reports of mortality rates more commonly associated with HPAIV outbreaks [44,55,80]. This is usually associated with confounding factors such as co-infection with bacterial or viral pathogens, and other factors such as poor nutrition and housing [81,82,83]. However, certain strains do also show high morbidity and mortality in controlled in vivo experiments [5,84,85,86,87].

Furthermore, when an HPAIV-like polybasic cleavage site was engineered into an H9 virus, an HPAIV phenotype was not observed in an H9N2 virus background. However, when the polybasic H9 HA was combined with the remaining genes from an HPAIV strain the reassortant virus did develop an HPAIV phenotype [88]. This implies H9N2 virus internal genes may not be compatible with an HPAIV phenotype in some cases.

### 3.2. H9N2 Virus Transmission and Host Tropism in Poultry

Four routes of transmission are widely described for influenza viruses: droplet, aerosol, faecal-oral and direct contact [89]. Droplet transmission describes exhaled particles >10 µm which are deposited into the upper respiratory tract, whereas aerosol droplets are typically less than 5 µm and can reach the lower respiratory tract [89]. Contact transmission relies on the transfer of particles to mucous membranes directly, or via a fomite intermediate. For a successful transmission event to occur, enough virus must persist long enough in the external environment to reach the target tissue. Transmission is therefore determined via several viral, host, and environmental aspects, including: (i) The major site of viral replication and viral titres shed; (ii) The distance and frequency between contacts and (iii) Environmental conditions and virus stability. In wild aquatic birds such as ducks and gulls AIVs generally exhibit gastrointestinal tropism and are thought to be spread primarily through the oral-faecal route. In poultry adapted AIVs, there exists some heterogeneity in tropism and transmission routes. HPAIV, such as H5N1, have a systemic distribution and are probably transmitted by a combination of the oral-faecal route and airborne transmission, whereas, LPAIVs in chickens tend to show more respiratory tropism, though some strains also show gastrointestinal tropism [16,84,89,90,91,92,93]. One of the key molecular markers that facilitates adaptation of an AIV from wild aquatic birds to poultry is the deletion of amino acids from the stalk domain of NA, which have been shown to mediate the switch to respiratory tropism in chickens [94,95]. There is good evidence to suggest that many LPAIV strains transmit by the airborne route, the oral-faecal route and the waterborne route [84,92,96]. However, the favoured mechanism of transmission between individuals varies by host species and viral strain. 

Many studies have implicated direct contact as an important transmission route for H9N2 viruses in chickens, although indirect routes such as aerosol and faecal-oral have been shown to be important for some strains and many viruses show primarily a respiratory tropism. However, some H9N2 strains have been shown to have an extended tropism for the kidneys or oviducts [97,98,99,100,101,102]. Both in the field and experimentally poultry adapted H9N2 viruses are mostly detected from buccal rather than cloacal swabs [77,84,103]. Additionally, inoculation of some H9N2 viruses into the respiratory tract is 40 times more effective than gastrointestinal inoculation at initiating infection [101]. However, many of these routes appear to be environmentally contextual, for example, at LBMs communal water sources have been implicated as the major route of transmission of endemic H5N1 and H9N2 viruses [77]. Together these studies indicate that for H9N2 and other enzootic poultry adapted H9N2 viruses, respiratory and contact transmission are likely the primary routes of transmission and that respiratory transmission may partly arise initially as an adaptation to poultry which clearly has implication for zoonotic transmission.

## 4. H9N2 Reassortment and Evolution

H9N2 viruses, although a threat in their own right, have been recognised recently as having donated gene segments to highly zoonotic viruses, therefore it is suggested that to prevent the emergence of new zoonotic viruses better control of H9N2 viruses is required [104].

### 4.1. H9N2 Viruses as Gene Donors

The 1997 HPAIV H5N1 outbreak in Hong Kong (the so-called clade zero viruses) has retrospectively been shown to have received its internal gene cassette (all genes except HA and NA) from co-circulating G1 lineage H9N2 viruses [10]. Genotype 57 (G57, also known as genotype S) viruses in China have recently become the predominant genotype circulating in poultry due to their enhanced fitness in poultry [16]. From 2013 onwards, reassortment between these G57 H9N2 viruses and other circulating subtypes resulted in the generation of multiple zoonotic AIVs with a high propensity to cause disease and death in humans as well as poultry such as: H7N9 [7], H10N8 [105] and, most recently, H5N6 all of which contain the six genes of the G57 internal gene cassette. Furthermore, several circulating HPAIV H5Nx viruses contain single or multiple genes from H9N2 [106,107,108], including the predominant genotype of H5 HPAIVs circulating in West Africa which contain a PB2 gene most likely donated from an H9N2 virus [109].

It has been shown, particularly for H7N9 viruses, that the G57 internal gene cassette greatly contributes to the pathogenicity of these viruses in mammals, again highlighting that the endemicity of H9N2 viruses may drive the emergence of future zoonotic influenza virus strains [104,110].

### 4.2. H9N2 Viruses asGene Recipients

As well as donating its entire internal gene cassette there have been multiple instances of H9N2 viruses receiving individual or multiple combinations of genes from other AIVs. For example, the predominant H9N2 lineage circulating Pakistan and Bangladesh is known to have received several genes from HPAIV H7N3 and H5N1 viruses [40,41,111]. Additionally, several Chinese H9N2 genotypes contain polymerase genes from H5N1 HPAIV [112].

There is evidence to suggest that these novel reassortant genotypes of H9N2 viruses, such as those found in Bangladesh, have become predominant due to higher fitness in poultry while also possessing a heightened zoonotic potential [102,113].

### 4.3. H9N2 Intrasubtypic Reassortment 

Overall, considering the large overlap and frequent co-infections between different influenza subtypes in chickens, intersubtypic reassortments remain rare. When intersubtypic reassortants are found, or experimentally generated, they rarely outcompete the currently circulating parental viruses to become the predominant genotypes (with the rare exceptions of the examples in the previous paragraphs) [40,109,114]. However, phylogenetic analysis suggests intrasubtypic reassortment (between different H9N2 viruses) occurs at a very high rate and has been shown to greatly contribute to the increasing fitness seen in these viruses in recent years [16,40,71]. This is likely due to the more similar host ranges, tropisms, and geographic spreads found between H9N2 viruses, as well as the fundamentally greater compatibility between gene segments that are more closely related to each other.

## 5. H9N2 Virus in Humans

### 5.1. History of Human Infections with H9N2

**Figure 2:**
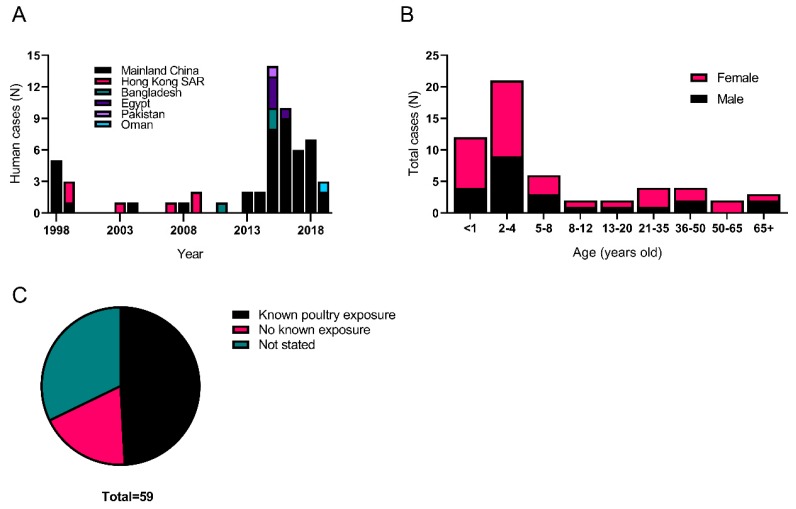
Distributions of human cases of H9N2 infection. (**A**) Laboratory confirmed cases over time and per country. (**B**) Age and Sex distribution of H9N2 infections. (**C**) Poultry exposure status of human H9N2 cases.

H9N2 viruses are fairly regularly isolated from humans, with the first reported human cases concerning two children, in Hong Kong in 1999, who exhibited flu-like symptoms. Retrospectively, several H9N2 infections on the Chinese mainland were also found to have occurred in 1998 [115,116]. Subsequent human infections have been reported from Egypt, Bangladesh, Pakistan and Oman [117,118,119,120]. Human H9N2 infections are generally mild and there has only been a single reported death due to the virus, likely due to an underlying health condition [121]. Human H9N2 cases are more often isolated during periods where other more pathogenic zoonotic influenza viruses are being surveyed for. Many H9N2 cases have been found recently in China, most likely due to the ongoing screening for zoonotic H7N9, and in Egypt and Bangladesh due to ongoing screening for zoonotic H5N1 infections (Figure 2a) [42,117]. As of June 2019, there have been a total of 59 laboratory-confirmed human H9N2 infections with over half of those being recorded since 2015 (see Table 2, Figure 2a). The majority of those with confirmed infections were young children (39 of 56 cases were aged 8 years or below, Figure 2b), the median age of infection was 4-years-old, while the mean age was 14. Both sexes appeared to get infected at similar rates (Figure 2b). This age and sex distribution is in stark contrast to that of the first wave of H7N9, which predominantly infected the elderly and males, and H5N1 viruses, which infected mostly young adults [122,123]. In the majority of infections, contact with poultry was confirmed as the likely source (29 with confirmed poultry exposure compared to 11 without any known poultry exposure, Figure 2c). However, unlike H7N9, there are still no confirmed reports of human-to-human transmission of H9N2 viruses [124]. Virus sequencing show that all human H9N2 isolates contain HA genes from the G1-W, G1-E or BJ94 lineages with virus isolates highly related to local poultry isolates [5,74,115].

### 5.2. Seropositivity Rates

The increase in H9N2 isolation rates due to greater screening of patients with influenza-like illness indicates that mild, or even symptomatic, human H9N2 cases may be relatively common. This possibility is supported by an extensive body of serological evidence showing particularly high seropositivity rates amongst poultry workers in many enzootic countries including India, Cambodia, China, Vietnam, Egypt, Hong Kong, Iran, Thailand and Pakistan (reviewed in [155]). Serological assays looking at H9 exposure suffer several limitations such as H9-antigenic cross-reactivity with other HA subtypes, however, in recent studies this limitation has been overcome through a number of approaches such as concurrent sero-typing against multiple human and avian HA subtypes, meta-analysis, and longitudinal studies of poultry workers [155,156]. Furthermore, there is a single study which has managed to isolate a virus from an asymptomatic poultry worker in Pakistan [118]. Overall this suggests that although H9N2 infections may be fairly common, they are mostly mild or asymptomatic and do not transmit any further than the initial zoonotic infection implying poor adaption of H9N2 viruses to mammals.

### 5.3. Haemagglutinin and Receptor Binding

Receptor binding preference of HA protein is a well-established determinant of zoonotic and pandemic potential [157,158]. Multiple studies have therefore attempted to evaluate this property of H9N2 AIVs. Initial studies showed that some H9N2 virus lineages, particularly the G1 and BJ94 lineages, appeared to possess a preference towards human-like α2,6-linked sialic acid over avian-like α2,3-linked SA. Subsequent studies utilised synthetic receptor analogues, including sulphated and fucosylated variants of the classically avian-like 3SLN receptor analogue. These studies showed that H9N2 viruses, particularly those of the G1 ‘Eastern’ sub-lineage and BJ94 lineage viruses, displayed high binding towards analogues sulphated on the antepenultimate sugar, though a few viruses of the G1 ‘Eastern’ sub-lineage also displayed moderate ‘human-like’ 6SLN binding [159,160]. A further study, utilising purified recombinant H9 HA and glycan arrays, found binding to α2,3-linked sialosides, as well as some binding to α2,6-, and α2,8- or α2,9- linked receptors [161]. Furthermore, several studies have looked at the receptor binding of BJ94 lineage viruses using ELISA based methods, these have unanimously showed that contemporary H9N2 viruses show a preference for the ‘human-like’ receptor analogue 6SLN over ‘avian-like’ 3SLN [162,163,164].

We speculate that many of the contemporary H9N2 viruses described as having a strong preference for human-like receptors likely possess a relatively much stronger preference towards sulphated avian-like receptors, and would suggest future studies utilise such analogues forthwith, in conjunction to classical 3SLN and 6SLN analogues.

#### Molecular Basis of Receptor Binding

Several studies have investigated the molecular basis of H9N2 receptor binding. In separate studies, it has been found that the HA receptor binding site residues 155, 190, 193, 226 and 227 (H3 numbering) are all involved in the receptor binding avidity of H9N2 viruses [162,163,165,166,167,168]. As with many other influenza subtypes, the substitution Q226L, appears to significantly shift the receptor binding of H9 HA towards a human-like preference in certain viral backgrounds [166]. However, there remains a need to better understand the molecular basis of receptor binding preference in H9N2 viruses to fully assess their zoonotic potential.

### 5.4. Ferret Experiments

Ferrets are considered the gold standard for assessing influenza virus zoonotic and pandemic potential in humans and have therefore been utilised to assess the intrinsic and adaptive potential of H9N2 viruses to infect and transmit between humans [169]. G1 lineage viruses have been tested for their ferret infectivity, as well as airborne and contact transmission several times. In three separate studies three different G1 ‘Eastern’ sub-lineage viruses and a single G1 ‘Western’ sub-lineage virus were shown to transmit efficiently to direct contact ferrets, but not via airborne transmission to sentinel ferrets [113,170,171]. Several BJ94 lineage viruses belonging to genotype 57, conversely, have been shown to be able to transmit, with varying degrees of efficiency, by respiratory droplet to contact ferrets [163,164]. Several studies have gone further and deliberately adapted H9N2 viruses to ferrets or made reassortants between H9N2 viruses and human strains and then tested these viruses for their infectivity and transmissibility in ferrets. A series of experiments by the Perez group took both these approaches. They initially showed that making a reassortant between a contact transmissible G1 ‘Eastern’ H9N2 virus and a human H3N2 virus was not enough to provide the virus with airborne transmissibility [170], therefore 10 ferret passages were performed. After 10 passages, respiratory droplet transmission between ferrets was achieved [172]. Furthermore, it was shown that an alternative reassortant containing the six internal genes from a 2009 pandemic H1N1 virus, and either the adapted, or unadapted H9N2 HA and NA were able to transmit between ferrets [173]. Overall, these studies indicate that H9N2 viruses are indeed viruses with pandemic potential, however, they would require some adaption and/or reassortment first to become a credible pandemic threat.

### 5.5. Other Factors Involved in Zoonotic and Pandemic Potential in H9N2 Viruses

Other than HA receptor binding several other factors have been well described as potentially giving H9N2 AIVs an intrinsic pandemic potential. HA pH stability is well described as being vital for adaptation of avian or swine influenza viruses to stable airborne transmission between ferrets or humans [157,158,174]. H9N2 viruses appear to have intrinsically more stable HAs compared to AIVs of the H5 and H7 subtype, in a similar range to early H1N1pdm09 viruses [160]. Furthermore, several adaptive mutations have been identified in field viruses that allow them to transmit by an airborne route between chickens, it is thought these would probably have the added effect of allowing more efficient transmission between humans as well [96,175].

## 6. H9N2 Infection in Other Species

Although the focus on H9N2 control and surveillance is largely on poultry and zoonotic infections there is a growing body of evidence of the virus in other species.

### 6.1. Minor Poultry Species

Although chickens appear to be the primary host for most poultry adapted H9N2 lineages, the virus is also endemic in minor poultry in many regions and appears to have evolved and adapted separately to members of these species, for example: quail, guinea fowl, partridge and pheasants [92,176]. The G1 ‘Eastern’ sub-lineage, in particular, appears to occupy a niche within these species [92,176]. Quail have been shown to possess a more ‘human-like’ receptor repertoire than chickens, containing a higher amount of α2,6-linked sialic acids [177,178], indicating that viruses adapted to these species may have a greater zoonotic potential than viruses circulating in chickens. This hypothesis is supported by the higher relative binding of viruses from this lineage to α2,6-linked receptor analogues, the higher replicative ability of these viruses in human primary tissues, and also by the higher than expected rate of zoonotic infections caused by these viruses, relative to their limited prevalence and geographical distribution [10,90,115,160,171,179]. Further, it has been shown that passage of a duck-origin H9N2 virus in quail leads to an expanded host range, with a virus that can more readily infect mice compared to the parental duck virus [180]. Poultry are also included in this host range expansion, which may explain the initial detection of an H9N2 virus in Japanese quail which preceded H9N2 establishment in poultry in endemic regions [100].

Due to the co-circulation G1 ‘Eastern’ sub-lineage and G57/H7N9 viruses, we hypothesise that a potential reassortment event between a naturally α2,6-binding G1 ‘Eastern’ virus and the naturally mammalian pre-adapted internal gene cassette of a G57-lineage virus could result in a virus with higher pandemic and zoonotic potential than either parental virus, therefore, continuous full genome surveillance of viruses, particularly in minor poultry, is vital in this region of Southern China.

### 6.2. Swine

Swine are often said to represent a potential ‘mixing vessel’ for human and avian viruses, a fact supported by semi-regular establishment of human and avian virus lineages in these hosts. There have been many recorded outbreaks of H9N2 virus in farmed pigs, mostly in Hong Kong and China [181,182,183,184]. As swine carry viruses closely related to human seasonal influenza viruses, it has been hypothesised a swine influenza/H9N2 reassortant could emerge with high pandemic potential [181]. Un-adapted, H9N2 viruses do not transmit efficiently between pigs, and swine H9N2 isolates show little evidence of mammalian adaption suggesting repeated reintroduction from avian hosts rather than continuous within-species circulation [184,185]. Repeated serial passage through pigs can lead to partial adaptation allowing for modest replication and transmission [185]. Although H9N2 viruses do not appear to actively circulate in pigs, there remains a possibility that these viruses could spill over into swine due to the proximity between poultry and pigs in many smallholding farms leading to the potential for reassortment with currently circulating swine influenza viruses.

### 6.3. Canids

Dogs are susceptible to several lineages of canine influenza viruses (CIV), the most common being equine-origin H3N8 and avian-origin H3N2 [186,187]. H9N2 viruses of the BJ94 lineage have been isolated in China several times from dogs with CIV-like illness [188], furthermore, a pair of studies have shown high seropositivity against H9 HA in stray dogs at LBMs in China, potentially due to feeding upon infected birds [189,190]. In 2016, a single avian-origin H3N2 CIV isolate was found that contained a PA gene closely related to that of circulating avian H9N2 viruses suggesting the possibility of active reassortment between AIV and CIV viruses in canine hosts [191]. Furthermore, there is serological evidence for H9N2 infection of foxes and racoon dogs in China, further indicating canids may be a potential host for these viruses [192].

### 6.4. Horses

Horses are hosts for several strains of equine influenza virus (EIV), most notably the currently circulating H3N8, and now extinct H7N7 strains. There is an isolated report of an H9N2 virus being isolated from a horse in Guanxi, China [193]. The virus was of the BJ94 lineage, the most common virus in poultry in the area, and most likely constituted a transmission event directly from poultry as no further, or follow up, cases were reported. However, as cases of equine influenza are rarely subtyped it is possible H9N2 viruses may be more common in these animals.

### 6.5. Mustelidae

As described earlier ferrets are a commonly used model for influenza virus infection and transmission due to their permissiveness to many different strains of influenza virus [169]. Mink, along with ferrets are members of the family *Mustelidae,* and are widely farmed for their fur. Like ferrets, farmed mink is susceptible to human and avian influenza viruses including H9N2 and there are several reports of H9N2 being isolated from farmed mink in China [192,194,195,196]. All isolates were of the BJ94 lineage prevalent throughout China. Interestingly two of the mink H9N2 isolates contained the mammalian adaptation in PB2, E627K, which is commonly seen during experimental adaptation of AIVs to ferrets [157,196]. Furthermore, several serosurveys have been performed on mink to look for the prevalence of anti-H9N2 antibodies, all three studies have shown a high seropositivity in farmed minks in China of between 20% and 45% [192,196,197]. Sea otters are also members of the family *Mustelidae,* a single serosurvey has found antibodies against H9 HA, however, this is perhaps unsurprising considering the presence of H9 viruses in seabirds and the relatively long lifespans of the otters [198].

### 6.6. Lagomorpha

Pikas are small rodent-like mammals of the order lagomorpha (which also includes rabbits.) There is evidence from serosurveys and from direct virus isolation that H9N2 viruses naturally infect pikas in China [199,200]. HA phylogeny of the pika isolates show these viruses are of the American lineage, known to occasionally infect wild birds in Asia [200]. As pikas are known to be able to be experimentally infected with avian influenza viruses, and due to the lack of any signature of mammalian adaptation (i.e., PB2 E627K), it appears more likely these infections are due to direct contact with infected birds or virus contaminated water sources rather than continuously circulating, mammalian adapted viruses (as may be the case with the H9N2 infected minks described in Section 6.5) [200,201].

### 6.7. Chiroptera

Recently there has been a single report of an H9N2-like virus isolated from bats in Egypt [202]. Unlike other bat influenza subtypes H17 and H18, the H9N2-like bat virus was able to be isolated in eggs and binds sialic acid as its receptor [1]. It does still appear though that, although the virus is highly divergent from all known avian H9N2 viruses, it was likely a recent (compared to H17 or H18) cross-species jump from birds followed by stable circulation in bats as the virus has several markers of mammalian adaptation such as PB2-D701N.

## 7. Vaccination and Control

Due to the economic damage caused by enzootic H9N2, many countries including China, Israel, South Korea, Morocco, Pakistan, Egypt, Iran and UAE have adopted vaccination at either a national or local level as a key approach for preventing H9N2 disease in poultry [30,44,52,203,204,205,206,207]. The most common vaccines in use are traditional inactivated vaccines, similar to those used in human seasonal vaccines. H9N2 viruses exhibit a wide antigenic variability, both between, and within lineages [10,16,168]. Unlike human vaccines, H9N2 vaccines are generally not as regularly assessed for their efficacy against antigenically drifted viruses and consequently are far less often updated. Therefore, in many regions H9N2 viruses continue to infect and cause disease in vaccinated poultry with tentative evidence suggesting that sub-optimal use of vaccination may be driving antigenic drift and/or clade replacement, and theoretically zoonotic potential and pathogenicity [16,29,30,49,103,206,208]. Because of this, there is a real need for: (i) better understanding of the molecular determinants of H9 antigenicity, (ii) better understanding of antigenic drift and the consequences upon viral fitness and zoonotic potential and (iii) next generation vaccines that protect against multiple strains and antigenically drifted variants.

Stamping out, which involves culling of potentially infected birds and birds presenting influenza-related morbidity has occasionally been used as a first line of defence against H9N2 in countries without a history of the virus. This was the case during early outbreaks in Korea and the recent outbreaks in Russia and Ghana [27,55,203]. However, once the virus becomes endemic in a country, stamping out becomes uneconomical and unfeasible, therefore vaccination is commonly used beyond this point. Stamping out is more often used during HPAIV outbreaks due to their status as notifiable diseases, regardless of a countries history with outbreaks/endemicity. 

Other than vaccination and stamping out, several other interventions have been successfully used in the field to halt or reduce avian influenza virus spread in poultry and subsequent zoonotic infection. As discussed above LBMs are a hotspot for influenza infection due to the convergence of a high density of different poultry species from across a wide geographic range. LBMs were identified early on as the main sources of AIV outbreaks in the late 1990s in China and Hong Kong and several interventions were utilised such as temporary closures, periodic rest days, and overnight market depopulation, as well as basic increases in biosecurity and hygiene practises. A detailed review of the effectiveness of these practises has previously been performed by Offeddu and colleagues, who concluded that these practises, particularly LBM closure, were effective at both halting the spread of AIV between birds, as well as having a knock-on effect at reducing zoonotic AIV cases [209]. A second detailed review by Fournié and colleagues indicated that individual as well as community-wide habits which expose humans to AIVs and risk of zoonotic infection are highly heterogeneous and may require control strategies tailored to individual communities [210].

## 8. Conclusions and Perspectives

In recent years, outbreaks of H9N2 viruses have been found in an increasing number of countries, including for the first time, sub-Saharan Africa, far South-East Asia and Russia. Because of its expansive geographical range, it is speculated that H9N2 viruses may currently be causing greater economic damage to poultry production worldwide compared to highly pathogenic H5 or H7 subtypes which are generally more localised. Moreover, the last four years have seen as many human H9N2 infections as the two decades before. These facts indicate a growing threat from H9N2 viruses to both animal and human health. Although the virus mostly causes mild disease and low mortality, as compared to highly pathogenic viruses, there is clear potential for the virus to continue to adapt and become more pathogenic in chickens and better adapted to humans. Additionally, there remains a clear threat, as highlighted by the repeated novel zoonotic AIV viruses that have emerged in recent years such as H7N9, H10N8 and H5N6, posed by reassortant H9N2-origin viruses.

H9N2 viruses have been repeatedly isolated from non-human mammalian hosts such as swine and minks—these hosts pose a particular threat for emergence of novel pandemic viruses as they are highly susceptible to both human and avian influenza viruses and could drive the generation of novel reassortants.

Endemic countries across Asia and the Middle East, as well as, more recently, Africa, are most under threat from zoonotic H9N2 infections. We have discussed how reassortants between H9N2 viruses and human seasonal influenza viruses are able to efficiently transmit between ferrets and there is, therefore, a real danger eventually such a reassortant could emerge in the field. Several H9N2 viruses have human receptor binding, pH stable HA proteins that could potentially allow efficient transmission between humans whilst other H9N2 viruses contain internal gene cassettes that allow extremely efficient replication in humans (i.e., genotype 57). Overall there is a clear risk of both intersubtypic H9N2/human influenza virus reassortant emergence as well as an intrasubtyptic human binding HA/efficient mammalian polymerase reassortant emergence, either of which could pose a high zoonotic and pandemic threat. 

These trends highlight a clear need for further surveillance efforts, particularly in countries where H9N2 has not been officially declared. Surveillance should also be continued in countries with endemic H9N2—in vaccinated poultry and poultry workers. Additionally, contemporary viruses circulating in poultry rearing systems need constant phenotypic characterisation to assess properties such as antigenic drift, viral pathogenicity and zoonotic potential.

## Figures and Tables

**Table 1:** List of countries with laboratory confirmed H9 infections in domestic gallinaceous poultry.

Country	Years of Poultry Isolates	Lineages	Species	Status	Recorded Human Cases/Serology
Afghanistan	2008–2009, 2016–2017	G1-W	Chicken	Potentially endemic	No
Algeria	2017	G1-W	Chicken	Potentially endemic	No
Bangladesh	2006–2007, 2009–present	G1-W, Y439	Chicken, Quail	Endemic	Virus isolated
Belgium	*1983* ^3^	Y439		H9N2-free	No
Burkina Faso	2017	G1-W	Chicken	Potentially endemic	No
Cambodia	2013, 2015, 2017–present	BJ94, G1-E, Y439	Chicken	Likely endemic	Serology only
Colombia	2005	n/a	Chicken	Unknown	No
China	1994–present	BJ94, G1-E, Y439	Chicken, Guinea Fowl, Quail, Partridge,	Endemic	Virus isolated and serology
Egypt	*2006*, 2011–2018	G1-W	Chicken, Quail, Turkey	Endemic	Virus isolated and serology
France	*1998, 2003*	Y439	Turkey	H9N2-free	No
Germany	*1994–1996*, *1998*^3^, *2004, 2012–2013*, *2015–2017*	Y439, G1-W	Chicken, Turkey	Recurrent infections from wild birds	No
Ghana	2017–present	G1-W	Chicken	Potentially endemic	No
Hong Kong SAR	*1988*, *1992*, *1994*, 1997, 1999–2000 2003, 2005–2012, 2014–2015	BJ94, G1-E, Y439	Chicken, Quail, Guinea Fowl, Partridge	Potentially endemic	Virus isolated and serology
Hungary	*2001*	Y439	Chicken	H9N2-free	No
India	2003–2004, 2006–2013, 2015	G1-W	Chicken	Potentially endemic	Serology only
Indonesia	*2002*, 2016–present	BJ94, Y439	Chicken	Likely endemic	No
Iran	1998–2017	G1-W	Chicken	Endemic	Serology only
Iraq	2005, 2008, 2014–2016	G1-W	Chicken	Potentially endemic	No
Israel ^1^	2000–2014, 2016–2017	G1-W	Chicken, Turkey, Ostrich	Potentially endemic	No
Italy	*1983–1985, 1989, 1994, 1996*	Y439	Chicken, Turkey	H9N2-free	No
Japan (Imported goods only)	1997, 2001–2002, 2015–2016	BJ94	Imported meat	H9N2-free	No
Jordan	2003–2007, 2010	G1-W	Chicken	Likely endemic	No
Kuwait	2003–2005, 2008	G1-W	Chicken	Potentially endemic	No
Laos ^2^	2009	n/a	Chickens	Potentially endemic	No
Lebanon	2004, 2010, 2017–present	G1-W	Chicken, Quail	Potentially endemic	No
Libya	2005, 2013	G1-W	Chicken	Potentially endemic	No
Malaysia	2018	n/a	n/a	Potentially endemic	No
Morocco	2016	G1-W	Chicken	Potentially endemic	No
Myanmar	2014–2015	BJ94	Chicken	Potentially endemic	No
Nepal	2009–2011	G1-W	Chicken	Potentially endemic	No
Netherlands	*2010–2011*	Y439	Chicken, Turkey	H9N2-free	No
Nigeria ^2^	2013	n/a	Chicken	Potentially endemic	Serology only
Oman	2006, 2019	G1-W	Chicken	Potentially endemic	Virus isolated
Pakistan	1998–2000, 2003–2012, 2014–2017, 2019	G1-W	Chicken, Partridge	Endemic	Virus isolated and serology
Poland	*2013–2014*	Y439	Turkey	H9N2-free	No
Qatar	2008	G1	Chicken	Potentially endemic	No
Romania ^2^	2009–2010	n/a	n/a	Unknown	Serology only
Russia (Eastern)	2018	G1-W, BJ94	Chicken	Unknown	No
Saudi Arabia	1998–2000, 2002, 2005––2008, 2010–2011, 2013, 2015–2016, 2018	G1-W	Chicken	Potentially endemic	No
South Africa	*1995, 2008–2009*	Y439	Ostrich	H9N2-free	No
South Korea	1996, 1999–2012	Y439(Korean)	Chicken, Guinea Fowl	Potentially endemic	No
Thailand ^2^	2008	n/a	n/a	Potentially endemic	Serology only
Tunisia	2010–2012, 2014	G1-W	Chicken, Turkey	Potentially endemic	No
USA	*1966, 1978, 1981, 1983, 1985, 1988–1989, 1993, 1995, 1997, 1999, 2001*	USA	Chicken, Quail, Turkey	H9N2-free	No
UAE	1999–2003, 2005–2006, 2008, 2011, 2015	G1-W	Chicken, Quail	Potentially endemic	No
Uganda	2017	G1-W	Chicken	Potentially endemic	No
UK	*1970, 2010, 2013*	Y439	Chicken, Turkey	H9N2-free	No
Vietnam	2009, 2012–2017	BJ94, G1-E, Y439	Chicken, Quail	Likely endemic	Serology only

^1^ Potential endemicity of Israel is based on apparent recurring epidemics, it is unclear how much is in situ circulation and how much is due to incursion from neighbouring countries. ^2^ Evidence for H9N2 virus in Nigeria, Romania, Thailand and Laos comes solely from sero-surveys. No viruses have been isolated from poultry/humans in these countries (though it is unclear whether any active surveillance has been performed that would detect H9N2). ^3^ Years where only viruses most likely transmitted directly from wild birds to poultry are shown in italics. All data provided in this table based on references used in this paper supplemented with sequences from GISAID (Global initiative on sharing all influenza data), NCBI (National Center for Biotechnology Information) influenza virus resource and FluDB databases as of June 2019 [31,32,33], see supplementary Table S1 for a full list of references and database accession numbers.

**Table 2:** Laboratory confirmed human cases of H9N2 infection.

Year	Location	Patient	Clinical Signs	Viral Lineage	Poultry Exposure?	Reference
1998	Guangdong province, China	14-year-old, male	ARI ^a^	BJ94	Yes, live chickens in dwelling	[116]
		75-year-old, male	ARI	BJ94	Yes, lived near farmers market	[116]
		4-year-old, male	ARI	BJ94	Unknown	[116]
		1-year-old, female	ARI	BJ94	Unknown	[116]
		36-year-old, female	ARI	BJ94	Yes, exposure to live poultry	[116]
1999	Guangdong province, China	22-month-old, female	Fever, cough	BJ94	No	[125]
	Hong Kong	13-month-old, female	Fever	G1 ‘Eastern’	Yes	[115]
		4-year-old, female	Fever, malaise	G1 ‘Eastern’	Unknown	[115]
2003	Hong Kong	5-year-old, male	Fever, cough	BJ94	No	[126]
2004	Guangdong Province, China	Unknown	Unknown	G1 ‘Eastern’	Unknown	[127]
2007	Hong Kong	9-month-old, female	Mild illness	ND ^c^	Yes	[128]
2008	Guangdong province, China	2-month-old, female	ILI ^b^	ND	Unknown	[128]
2009	Hong Kong	35-month-old, female	Fever, cough,	G1 ‘Eastern’	Unknown	[129]
		47-year-old, female	Fever, cough	G1 ‘Eastern’	No	[130]
2011	Dhaka, Bangladesh	4-year-old, female	Fever, cough	G1 ‘Western’	Yes, close exposure to sick poultry	[117]
2013	Guangdong province, China	86-year-old, male	Cough	BJ94	No	[131]
	Hunan province, China	7-year-old, male	Fever	BJ94	Yes, close contact to poultry	[74,131]
2014	Sichuan Province, China	2.5-year-old, male	Mild illness	BJ94	Unknown	[119,132]
	Guangdong province, China	Unknown	Mild illness	BJ94	Unknown	[119]
2015	Aswan, Egypt	3-year-old, male	Unknown	ND	Yes	[119]
	Cairo, Egypt	7-year-old, female	ILI	ND	Yes	[119]
		9-month-old, female	ILI	ND	Yes	[119]
	Guangdong province, China	male	Unknown	BJ94	Unknown	[133]
	Bangladesh	3.5-year-old, female	Mild illness	ND	Yes, close contact with sick poultry	[134]
	Anhui province, China	4-year-old, female	Mild illness	BJ94	Yes, live bird market exposure	[135]
	Hunan province, China	2-year-old, male	Mild illness	BJ94	Unknown	[75,135]
	Anhui province, China	6-year-old, male	Unknown	BJ94	Unknown	[136]
	Hunan province, China	15-year-old, female	Mild illness	ND	No	[75,135]
		11-month-old, female	Mild illness	ND	No	[135]
	Dhaka, Bangladesh	46-year-old, male	Fever	ND	Yes, poultry worker, exposure to sick birds	[137]
	Guangdong province, China	84-year-old, female	Unknown	ND	Unknown	[121]
	Punjab district, Pakistan	36-year-old, male	Non-symptomatic	G1 ‘Western’	Yes	[118]
	Hunan province, China	2-year-old, male	Mild illness	BJ94	Yes, live bird market exposure	[138]
2016	Sichuan Province, China	57-year-old, female	ARI, Died ^d^	ND	Unknown	[139]
	Cairo, Egypt	18-month-old, male	ILI	ND	Yes, exposure to live bird market	[140]
	Guangdong province, China	4-year-old, female	ARI	BJ94	Yes	[141,142]
		29-year-old, female	ARI	ND	Unknown	[121]
	Yunnan province, China	10-month-old, male	ILI	ND	Yes	[121]
	Jiangxi province, China	4-year-old, female	Mild illness	ND	Unknown	[121]
	Henan province, China	5-year-old, female	Unknown	ND	No	[121]
	Guangdong province, China	3-year-old, male	Unknown	ND	Yes	[121]
	Guangdong province, China	7-month-old, female	Mild illness	ND	Yes	[143]
	Beijing, China	4-month-old, male	Mild illness	BJ94	Yes	[144]
2017	Gansu province, China	11-month-old, male	Mild illness	ND	Yes	[145]
	Beijing, China	32-year-old, male	Mild illness	BJ94	No	[144,146]
	Guangdong province, China	2-month-old, female	ILI	ND	Yes, poultry at home	[147]
	Hunan province, China	20-month-old, female	n/a	BJ94	Unknown	[148]
		9-month-old, male	ILI	BJ94	Unknown	[75,148]
	Anhui province, China	9-year-old, female	Mild illness	BJ94	Unknown	[149]
2018	Guangdong province, China	3-year-old, female	Mild illness	ND	Yes, exposure to live bird market	[149]
	Beijing, China	51-year-old, female	Mild illness	ND	Yes, exposure to slaughtered poultry	[149]
	Guangdong province, China	24-year-old, female (pregnant)	Mild illness	ND	Yes, exposure to farm	[150]
	Guangdong province, China	10-month-old, female	Mild illness	ND	Yes, backyard poultry exposure	[151]
	Guangxi province, China	3-year-old, male	n/a	BJ94	No	[151]
	Guangdong province, China	32-year-old, female	Pneumonia	ND	Unknown	[152]
	Hunan province, China	2-year-old, male	Mild illness	BJ94	No	[153]
2019	Yunnan province, China	8-year-old, female	Mild illness	ND	No	[153]
	Jiangsu province, China	9-year-old, male	Severe pneumonia	ND	Yes	[154]
	Oman	13-month-old, female	ILI	G1 ‘Western’	Yes	[120]

^a^ ARI—acute respiratory infection. ^b^ ILI—influenza-like illness. ^c^ ND—strain lineage not reported. ^d^ Underlying health conditions were cited as contributing factor.

## Supplementary Materials

The following are available online at https://www.mdpi.com/1999-4915/11/7/620/s1. Table S1: Evidence of H9N2 viruses in poultry in different countries and different species.
